# New NADPH Oxidase 2 Inhibitors Display Potent Activity against Oxidative Stress by Targeting p22^phox^-p47^phox^ Interactions

**DOI:** 10.3390/antiox12071441

**Published:** 2023-07-18

**Authors:** Adriana V. Treuer, Mario Faúndez, Roberto Ebensperger, Erwin Hovelmeyer, Ariela Vergara-Jaque, Yunier Perera-Sardiña, Margarita Gutierrez, Roberto Fuentealba, Daniel R. González

**Affiliations:** 1 Department of Basic Biomedical Sciences, School of Health Sciences, Universidad de Talca, Avenida Lircay s/n, Talca 3460000, Chile; 2 Departamento de Farmacia, Escuela de Química y Farmacia, Facultad de Química y de Farmacia, Pontificia Universidad Católica de Chile, Av. Vicuña Mackenna 4860, Santiago 7820436, Chile; 3 Escuela de Química y Farmacia, Facultad de Medicina y Ciencia, Universidad San Sebastián, Santiago 7510157, Chile; 4 Center for Bioinformatics, Simulation and Modeling, Faculty of Engineering, Universidad de Talca, Avenida Lircay s/n, Talca 3460000, Chile; 5 Organic Synthesis Laboratory and Biological Activity (LSO-Act-Bio), Institute of Chemistry of Natural Resources, Universidad de Talca, Talca 3460000, Chile; 6 Escuela de Enfermería, Facultad de Salud, Universidad Santo Tomás, Talca 3460000, Chile

**Keywords:** heteroaryl-acrylonitrile, NOX inhibitors, HL-60 cells, p47^phox^, reactive oxygen species, mdx

## Abstract

NADPH oxidase (NOX2) is responsible for reactive oxygen species (ROS) production in neutrophils and has been recognized as a key mediator in inflammatory and cardiovascular pathologies. Nevertheless, there is a lack of specific NOX2 pharmacological inhibitors. In medicinal chemistry, heterocyclic compounds are essential scaffolds for drug design, and among them, indole is a very versatile pharmacophore. We tested the hypothesis that indole heteroaryl-acrylonitrile derivatives may serve as NOX2 inhibitors by evaluating the capacity of 19 of these molecules to inhibit NOX2-derived ROS production in human neutrophils (HL-60 cells). Of these compounds, **C6** and **C14** exhibited concentration-dependent inhibition of NOX2 (IC_50_~1 µM). These molecules also reduced NOX2-derived oxidative stress in cardiomyocytes and prevented cardiac damage induced by ischemia-reperfusion. Compound **C6** significantly reduced the membrane translocation of p47^phox^, a cytosolic subunit that is required for NOX2 activation. Molecular docking analyses of the binding modes of these molecules with p47^phox^ indicated that **C6** and **C14** interact with specific residues in the inner part of the groove of p47^phox^, the binding cavity for p22^phox^. This combination of methods showed that novel indole heteroaryl acrylonitriles represent interesting lead compounds for developing specific and potent NOX2 inhibitors.

## 1. Introduction

The nicotinamide adenine dinucleotide phosphate (NADPH) oxidase (NOX) is a unique enzymatic system whose sole activity is the production of superoxide (O_2_^•−^) and hydrogen peroxide [1]. This multicomponent enzyme catalyzes the reduction of molecular oxygen (O_2_) to O_2_^•−^, with NADPH as an electron donor, through the transmembrane protein cytochrome b558, a heterodimeric complex of two proteins, gp91^phox^, and p22^phox^ [2]. 

Homologues of the phagocytic cytochrome b558 (NOX2/gp91^phox^) subunits are NOX1, NOX3, NOX4, NOX5, DUOX1, and DUOX2 [3]. They are found in different tissues, acting as rich sources of reactive oxygen species (ROS), presenting important differences in mechanisms of activation and their physiological functions [4]. 

As mentioned, NOXs are enzymatic complexes with a transmembrane catalytic subunit, encompassing cytochrome b558, composed of a large gp91^phox^ subunit and a p22^phox^ subunit. In the case of NOX2, the cytoplasmic proteins p47^phox^, p40^phox^, and p67^phox^ and a small GTPase Rac are required for the activation of the complex and enzymatic activity [5]. 

The activation of NOX2 occurs after the association of the cytoplasmic subunits, p47^phox^, p67^phox^, and p40^phox^, to the membrane-bound gp91^phox^ catalytic domain through the activity of Rac [6]. The p47^phox^ subunit contains an N-terminal phox (PX) domain, which is a target for the action of PIP3, while the C-terminus of p47^phox^ contains serine-rich sites, which are a target for phosphorylation by PKC and MAPK [7]. Upon phosphorylation, the cytoplasmic subunits p47^phox^ and p67^phox^ promote the binding of p40^phox^ to p47^phox^ [8]. In addition, phosphorylation of the p47^phox^ and p67^phox^ subunits promotes their translocation to the cell membrane, binding to the catalytic subunit and allowing direct association with p22^phox^, facilitating the binding of p67^phox^ and p40^phox^ to cytochrome b558, the membrane-bound hetero-dimeric complex formed by the gp91^phox^ and p22^phox^ [9]. 

NOX2 is responsible for the production of ROS in neutrophils, which is part of the defensive mechanisms of the innate immune system against pathogens [10,11]. Although the physiological production of ROS plays an important role in redox signaling in several systems, in pathological conditions NOXs are responsible for an increased level of ROS, producing oxidative stress [12]. High levels of ROS cause structural tissue damage, which has been associated with the development of several pathological states, such as diabetes and cardiovascular diseases [13,14,15]. For this reason, there is substantial interest in the discovery and development of pharmacological NOX inhibitors in order to regulate this redox imbalance.

Considering the role of NOXs in oxidative stress and their effect on various pathologies such as immunosuppression [16], neurodegeneration [17], and cardiovascular diseases [18], the need to develop new and selective inhibitors of NOXS has emerged.

A wide variety of chemical compounds have been evaluated as NOX inhibitors with the idea of protecting the body against ROS-associated pathologies. Among these substances, the first NOX2 inhibitors developed were diphenyleneiodonium (DPI) and apocynin, which are unspecific [19]. Later, VAS2870 [20] and GSK2795039 [21], to mention some of the most important, were developed. Since there are no specific inhibitors for NOX2 so far, it is necessary to develop new compounds that can decrease or inhibit its activity and hence ROS production [22]. 

Structurally, these NOX inhibitors are mostly planar, aromatic heterocyclic molecules. Among heterocycles, there is an outstanding group of highly reactive scaffolds, such as quinolone [23], isoxazole [24], pyrazole [25,26], imidazole [27], pyridine [28], pyrimidine [29], and the indole nucleus [30]. These pharmacophores have received special attention within the field of medicinal chemistry due to their versatile reactivity to design synthetic hybrids with potential applications in drug design. 

In particular, indole derivatives have been one of the most studied scaffolds to date in the chemistry of heterocyclic compounds, and they are found in various pharmacologically and biologically active compounds with a wide range of pharmacological properties, such as anti-malarial [31], analgesic [32], hepatitis C virus inhibitors [33], A2 phospholipase inhibitors [34], anti-carcinogenic [35], anti-fungal and antimycobacterial [36], anti-tubulin agents [37], and protein kinase inhibitors [38].

In addition, the indole nucleus has been shown to serve as a precursor of several derivatives, including cyanoacetylindole, a versatile nucleophile, since it is an activated methylene system, which has been reported as a building block to prepare a wide variety of alkenes functionalized through the Knoevenagel reaction for the synthesis of heteroaryl-acrylonitrile derivatives [39,40,41]. The acrylonitrile group, an α, β-unsaturated nitrile, is able to increase the solubility of a compound and is a potential site to establish hydrogen bonds and hydrophobic interactions with target proteins [42].

Heteroaryl-acrylonitrile derivatives are known to have interesting pharmacological properties, such as antihyperglicemic [43], antifungal [36], antitumor [44], antibacterial, and cytotoxic [45,46], as well as acetylcholinesterase inhibitors [39], kinase-3 inhibitors (JNK3) [47], and tubulin inhibitors [48]. 

Here we aim to demonstrate the hypothesis that (*E*)-2-(1*H*-Indole-3-ylcarbonyl)-3-heteroaryl-acrylonitrile derivatives may serve as potential NOX2 inhibitors based on their structure, where the indole group is a pharmacophore of interest. For this, we evaluated a series of 19 indole heteroaryl-acrylonitriles that were synthesized in our group and tested them through different assays. The data obtained revealed that two of these compounds showed potent NOX2 inhibitory activity.

## 2. Materials and Methods

### 2.1. Indole Heteroaryl-Acrylonitriles Synthesis

Nineteen indole heteroaryl-acrylonitriles were synthesized from cyanoacetylindole and a series of aromatic aldehydes in ethanol through the microwave-assisted Knoevenagel condensation reaction. Compounds were purified and characterized as previously reported [40,49].

### 2.2. Cells and Reagents 

Cell culture medium (RPMI-1640) and fetal bovine serum (FBS) were purchased from Biological Industries (Kibbutz Beit Haemek, Israel). Penicillin/streptomycin were obtained from Hyclone, Logan, UT (USA). 7-(1,3-Benzoxazol-2-ylsulfanyl)-3-benzyl-3H-[1,2,3]triazolo [4,5-d]pyrimidine (VAS2870) was purchased from Calbiochem San Diego, CA (USA). Lucigenin was obtained from Roche (Little Falls NJ, USA). Etoposide, 3-(4,5-Dimethyl-2-thiazolyl)-2,5-diphenyl-2H-tetrazolium bromide (MTT), dimethylsulfoxide (DMSO), phorbol myristate acetate (PMA),2,2-diphenyl-1-picrylhydrazyl hydrazide (DPPH), sodium dodecyl sulfate (SDS), quercetin, catechin, 2,3-butanedione monoxime (BDM), taurine, nicotinamide adenine dinucleotide phosphate reduced (NADPH), ethylene glycol bis(β-aminoethylether)-N,N,N’,N’-tetraacetic acid (EGTA), phenylmethylsulfonyl fluoride (PMSF), cytochrome C, potassium cyanide (KCN), and all other chemicals were purchased from Sigma-Aldrich (St. Louis, MO, USA).

### 2.3. HL-60 Cell Differentiation

The human promyelocytic leukemia cell line HL-60 (ATCC-No.CCL 240, obtained from The European Collection of Authenticated Cell Cultures (ECACC)) was used to differentiate neutrophils. HL-60 cells were grown in 75 cm^2^ bottles in a humidified atmosphere with 5% CO_2_ in RPMI 1640 culture medium supplemented with 10% FBS and 100 UI/mL penicillin-streptomycin, replenishing the culture medium every 2 days.

The differentiation of HL-60 cells to neutrophils was performed after reaching a concentration of between 2 × 10^6^ and 5 × 10^6^ cells/mL in the differentiation medium: RPMI 1640 medium; 10% FBS; 100 IU/mL of penicillin/streptomycin; and 1.3% DMSO for 7 days, replenishing the differentiation medium every 2 days.

### 2.4. Cytotoxicity Assays

To verify the cytotoxicity of the acrylonitrile derivatives, the MTT reduction method was performed. Briefly, HL-60 cells were seeded in a flat-bottomed 96-well plate at a density of 10,000 cells per well. Then, the cells were first incubated with the synthesized compounds at different concentrations (1 µM, 10 µM, 100 µM, 1 mM, and 10 mM) in 200 μL of 10% fetal bovine serum-RPMI culture medium at 37 °C for 24 h. In a second series of experiments, cells were incubated under the same conditions but for 72 h. Etoposide was used as a positive control. Ten μL of MTT was added at a final concentration of 0.5 mg/mL, incubated at 37 °C for 4 h, and then solubilized with 10% SDS in 0.1 mM HCl and incubated overnight at 37 °C. Finally, a blue-colored compound (formazan) formation was measured at 570 nm in a multiplate reader (StatFax 4200, Awareness Technology, Inc., Palm City, FL, USA). The viability percentage was obtained as follows: Viability % = [treated cells DO × 100]/[DO cells control].

### 2.5. Cardiomyocyte Isolation from Mdx Mice

Cardiomyocytes were isolated from *mdx* mice (C57BL/10ScSn-DMDmdx/J, stock number 001801M, Jackson Laboratories, Bar Harbor, MA, USA). Male mdx mice of 19 months of age were used, which were housed in individual cages with water and food ad libitum. All the procedures were adjusted to the “Guide for the Care and Use of Laboratory Animals” (1996) of the National Institute of Health and were approved by the Institutional Committee for Animal Care and Use of the Universidad de Talca. Mice were pre-medicated with heparin i.p. (200U.) and then sacrificed by cervical dislocation. The heart was rapidly removed, cannulated through the aorta, and retrogradely perfused in a steady flow (2 mL/min) at 37 °C. The hearts were initially perfused for 10 min with a Ca^2+^-free bicarbonate buffer solution containing (in mM) 120 NaCl; 5.4 KCl; 1.2 MgSO_4_; 1.2 NaH_2_PO_4_; 5.6 glucose; 20 NaHCO_3_; 10 BDM, and 5 taurine, bubbled with a 95% O_2_–5% CO_2_ mixture. Enzyme digestion was performed with collagenase type II (1 mg/mL; Worthington Biochemical Corp., Lakewood, NJ, USA) and protease type XIV (0.1 mg/mL; Sigma-Aldrich) as previously reported [50]. Isolated cardiomyocytes were transferred to 1.5 mL Eppendorf tubes and preincubated with the heteroaryl acrylonitrile compounds.

### 2.6. Isolated Rat Heart Preparation

Male Sprague–Dawley rats (200–300 g) were obtained from the animal facility of the Universidad de Talca. Animals were kept at 22 °C with water and food (Rat Diet 5012, Lab Diet) ad libitum. The experimental protocols were approved by the Universidad de Talca Institutional Animal Care Committee (protocol # 2017-13-A) in accordance with the Guide for the Care and Use of Laboratory Animals published by the US National Institutes of Health (NIH Publication No. 85–23, revised 1996). The animals were anesthetized with sodium thiopental (Provet, Santiago, Chile), 10 mg/kg i.p., and pre-medicated with heparin (Laboratorio Biosano, Santiago, Chile), 1000 UI i.p. With the animals under deep anesthesia, hearts were rapidly excised and cannulated through the aorta and placed in a heated chamber for perfusion with Krebs–Henseleit buffer, containing (in mM): NaCl 118.5, NaHCO_3_ 25, KCl 4.7, MgSO_4_ 1.2, K_2_HPO_4_ 1.2, CaCl_2_ 2.5, and glucose 11, equilibrated with a gas mixture of 95% O_2_–5% CO_2_, at 37 °C, using a Master Flex peristaltic pump (Cole-Parmer, Barrington, IL, USA), as previously described [51]. A polyvinyl chloride balloon is connected to a pressure transducer by a polyethylene P-50 cannula placed through the left atrium and mitral valve into the left ventricle. The balloon was filled with saline to determine isovolumetric intraventricular pressure. Perfusion flow was set at 10 mL/min and kept constant. Left ventricular pressure (LVP) and coronary perfusion pressure (CPP) were monitored continuously with pressure transducers (P23XL, Ohmeda Instruments, Madison, WI, USA) and digitized (Chart, ADI Instruments, New South Wales, NSW, Australia).

After cannulation, hearts were perfused with Krebs–Henseleit solution and allowed for 15 min of equilibration. Then, the hearts were perfused with Krebs-Henseleit solution containing either vehicle (5% dimethyl sulphoxide, DMSO) or **C6** or **C14**, 1 µM, throughout the experimental protocol: after an equilibration post cannulation, hearts were perfused for 15 min for baseline, then flow was stopped, and ischemia proceeded for 45 min. After this period, perfusion was re-initiated and lasted for 60 min.

### 2.7. Infarcted Area Calculation

Following the ischemia-reperfusion protocol, the hearts were perfused with a solution containing 1% (2,3,5)-triphenyltetrazolium (TTC) dissolved in phosphate buffer saline (PBS) at 37 °C for 15 min. Then the hearts were removed, placed in a metallic cast (Roboz, Gaithersburg, MD, USA), and cut into 2–3 mm slices with a microtome blade. Five slices were photographed on both sides with a Nikon D3300 digital camera (Nikon Inc., Tokyo, Japan). The infarcted area was calculated using the NIH Image J software (ImageJ/Fiji 1.46, https://imagej.nih.gov/ij/index.html, accessed on 9 May 2023) by the following calculation: TTC-excluded areas on each side of each slice were averaged to get a mean infarct area per slice. This number was divided by the total slice area to obtain the percentage infarct area.

### 2.8. Preparation of HL-60 Cell Membranes

Neutrophil-differentiated HL-60 cells (2 × 10^6^ cells/mL) were incubated at 37 °C in a humid atmosphere with 5% CO_2_ and 50 μM of the different acrylonitrile compounds, then PMA 0.8 μM was added and incubated for 1 h to induce the activation of the enzyme NADPH oxidase. As a positive inhibition control, VAS2870 (20 μM) was used. Another two controls were performed: one with cells activated by PMA in the presence of DMSO, and the other (NA, non-activated) with cells in the absence of PMA and the acrylonitrile compounds. In 15 mL conical tubes, 5 mL of cell suspension from each treatment and control condition was transferred at 2 × 10^6^ cells/mL and centrifuged at 1000 rpm at 4 °C for 10 min. The supernatant was then discarded, and each pellet was re-suspended in 2 mL of a 4% PBS solution with 2 mM EGTA and 0.25 mM PMSF frozen at −80 °C for 10 min. Then each pellet was thawed on ice. After this, the cell suspension was passed through a glass tissue homogenizer (Deltalab^®^, I.C.T, S.L, La Rioja, Spain) 20 times. The freezing-thawing and homogenization processes were repeated three times. Finally, the homogenate was centrifuged at 15,000 rpm at 4 °C for 15 min. The supernatant (cytosolic fraction) was removed from the pellet (membranes were prepared). A fraction was removed for the determination of total proteins, and the remainder was stored at −80 °C for further analysis.

### 2.9. NADPH Oxidase Activity

The chemiluminescence analysis of lucigenin derivatives was used to determine the activity of NADPH oxidase in a suspension of membrane preparations of HL-60 cells. For this, 100 mg of total protein from the suspensions of the membrane preparations were placed in a 96-well microplate at a final volume of 200 µL with PBS and incubated at 37 °C for 30 min with the acrylonitrile derivatives. VAS2870 (20 µM) was used as a control of inhibition. Chemiluminescence was measured for 1 h at 2 min intervals with a Synnergy H1 hybrid luminometer multi-mode microplate reader and GEN5 program (Biotek Instruments, Inc., Winooski, VT, USA). The reaction started with the addition of 100 μM NADPH to the cell suspension (200 μL: 100 μg protein + PBS) containing 100 μM lucigenin. The activity was expressed as relative units of luminescence per second, relative to the control.

### 2.10. Cytochrome C Reduction

The membrane preparations from the neutrophil-differentiated HL-60 cells, treated with the different indole heteroaryl-acrylonitrile derivatives, were re-suspended in PBS with 10 μL/mL of the protease inhibitor cocktail (in mM): 104 (4-(2-aminoethyl)benzenesulfonylfluoride hydrochloride); 1.4 N-(trans-epoxysuccinyl)-L-leucine-4-guanidinobutyric; 2 leupeptin hemisulfate; 4 bestatin hydrochloride; 1.5 pepstatin A; and 80 μM aprotinin. Total proteins were quantified by the bicinchoninic acid (BCA) method using the commercial kit BCA™ (Thermo Scientific Pierce™, Rockford, IL, USA), using BSA as a standard. In brief, 100 μg of each homogenate were placed in a 96-well microplate, completing a 300 μL final volume per well with 36 μM cytochrome C in potassium phosphate buffer solution (in mM): 300 K_3_PO_4_, pH 7.8; 0.1 EDTA, 1 KCN in ultra-pure water, and 100 μM NADPH were added prior to reading for 30 min. The reaction was monitored at 550 nm for 30 min at 25 °C using PBS as a blank.

### 2.11. NADPH Oxidase Inhibition Protocol in HL-60 Cell-Derived Neutrophils

The 2′,7′-dichlorofluorescein (DCF) fluorescence analysis was used to determine NADPH oxidase activity in neutrophil-differentiated HL-60 cells. The method, based on a fluorimetric assay, was adapted from Boly et al. [52]. Briefly, 1 × 10^6^ neutrophil-differentiated HL-60 cells/mL were incubated for 45 min. at 37 °C in darkness with DCF-DA (1 mg/mL). The cell suspension was then transferred to a 15 mL conical tube and centrifuged at 330× *g* × 10 min at 37 °C. After the supernatant was removed, the cells were re-suspended in Hanks balanced saline buffer (HBSS, Sigma-Aldrich, St. Louis, MO, USA) and transferred to dark 96-well plates with a flat glass bottom (Thermo Scientific Nunc 96 microwell^®^ black, Optical Botton # 1.5 Cover Glass Base, Thermo Fisher Scientific). Then, 200 μL of cell suspension per well were incubated at 37 °C for 30 min with the indole heteroaryl-acrylonitriles at increasing concentrations (0.05 to 50 μM). As a positive control for the inhibition of NADPH oxidase, VAS2870 (20 μM) was used. Finally, PMA (0.8 μM) was added to each well for 10 min. to generate the activation of NOX2. The generation of the DCF fluorescence product was monitored in an automatic microplate reader (FLx800, BioTek Instruments, Winooski, VT, USA) at 1-min intervals at 37 °C for 1 h, using an excitation wavelength of 485 nm and an emission wavelength of 555 nm. The ability to inhibit ROS production by activated neutrophil-differentiated HL-60 cells was compared against the different controls. A control was performed with cells activated by PMA in the presence of DMSO and established as having 100% of the ROS-induced fluorescence. Inhibition control was performed in the presence of VAS2870 (20 µM) and activated by PMA. Another control (NA, not activated) was done with cells in the absence of PMA and the indole heteroaryl-acrylonitriles.

### 2.12. Assessment of DPPH Radical Scavenging Activity

The newly synthesized compounds were evaluated for 2,2-diphenyl-1-picrylhydrazyl hydrazide (DPPH^+^) free radical scavenging activity. The reduction and stabilization of DPPH by antioxidant compounds lead to discoloration, resulting in a decrease in absorbance at 517 nm, which was expressed as a percentage of the radical solution at a given concentration of discoloration. The heteroarylacrylonitrile derivatives were re-suspended in a minimum of DMSO at a final concentration of 5 mg/mL (stock solution). From these, they were diluted to concentrations of 1000, 500, and 100 μg/mL. These solutions were mixed with a methanol solution of DPPH (20 μg/mL) for 5 min. Each compound was evaluated in triplicate; absorbance at 517 nm in a UNICAM UV/visible spectrophotometer Helios-α (Thermo Electron Corporation, Waltham, MA USA) was measured by subtracting the solvent absorbance and comparing the resulting value with that of a blank sample. The percentage of discoloration was established according to the following formula: % Radical removal or discoloration = (1 − (AM − ABM)/AC) × 100, where AM is the absorbance of the sample compound, ABM is the absorbance of the sample blank, and AC is the absorbance of the control. The degree of discoloration indicates the efficacy of the compounds studied as free radical scavengers; therefore, a percentage of discoloration of 100 indicates the maximum radical scavenging capacity and a value close to 0 indicates a zero radical scavenging capacity. Quercetin was used as a reference compound in a concentration range of 1.25 to 7.5 μg/mL.

### 2.13. NOX2-Mediated ROS Production Inhibition in Mdx Cardiomyocytes

Cardiomyocytes isolated from mdx mice were transferred to Eppendorf tubes to be incubated with 1 μM of the indole heteroaryl-acrylonitrile derivatives for 30 min, protected from light, at room temperature. VAS2870 (20 μM) was used as a positive control for NOX2 inhibition. 2′,7′-dichlorofluorescein diacetate (DCF-DA) (10 μM) was then added and incubated for 15 min. Subsequently, it was allowed to sediment by gravity to remove the supernatant, and the pellets obtained were in a phosphate saline solution (PBS). 200 μL of the cell suspension were placed on pre-treated polylysine slides (Thermo Fischer, USA) and fixed with 2% paraformaldehyde (Sigma-Aldrich^®^, St. Louis, MO, USA) for 10 min at 4 °C. The preparations were then washed with PBS three times and assembled in a ProlongGold medium (Invitrogen, Carlsbad, CA, USA). Finally, the preparations were observed by the confocal microscope LSM700 (Carl Zeiss Microscopy, Oberkochen, Germany) with an excitation wavelength of 495 nm and an emission wavelength of 527 nm. The experiments were performed in triplicate, and a minimum of six fields per slide per condition were observed. From the acquired images, the fluorescence intensity was quantified at 495 nm, and the data were reported as fluorescence intensity per pixel.

### 2.14. Immunofluorescence and Confocal Microscopy

Isolated cardiomyocytes and neutrophil-differentiated HL-60 cells were prepared as described above. A total of 200 μL of cell suspension were placed on pre-treated polylysine slides (Thermo Fischer, USA) and fixed with 2% paraformaldehyde (Sigma-Aldrich^®^, St. Louis, MO, USA) for 10 min. at 4 °C. The preparations were then washed with PBS three times and incubated overnight at 4 °C with the primary monoclonal antibodies against the NADPH oxidase subunits: NOX2 (gp91^phox^) and p67^phox^ (BD Biosciences, Franklin Lakes, NJ, USA), p22^phox^ (Santa Cruz Biotechnology, Santa Cruz, CA, USA), and p47^phox^ (Upstate, Lake Placid, NY, USA). The next day, the preparations were washed with PBS and incubated at 37 °C for 1 h with the anti-mouse-TRITC and anti-rabbit-FITC secondary fluorescent antibodies (Jackson ImmunoResearch, West Grove, PA, USA). Finally, the preparations were washed to remove excess fluorescent antibodies and mounted on a ProlongGold medium (Invitrogen, USA). The images were obtained using a Zeiss LSM-700 confocal microscope (Carl Zeiss Microscopy, Germany) with 40× magnification.

### 2.15. Preparation of Protein Extracts, and Quantification of Total Proteins

Neutrophil-differentiated HL-60 cells and/or cardiomyocytes were homogenized in lysis buffer (in mM): 50 Tris; 30 NaCl; 2 EDTA, 0.1% SDS, and Protein Inhibitor Cocktail (Complete Mini EDTA-free, Roche, USA) using T 10 basic Ultra-Turrax^®^ in speed scale 4 (approx. 22,000 rpm). Each sample was then centrifuged at 13,200 rpm for 15 min at 4 °C; the pellet was discarded, and the supernatant was stored for future analysis.

The total protein was quantified by the bicinchonic acid method (BCA), using the commercial BCA ™ kit (Thermo Scientific Pierce ™, Rockford, IL, USA), using BSA as standard. Briefly, the assay was performed following the manufacturer’s instructions for the microplate reading protocol (Thermo Scientific Pierce BCA Protein Assay Kit, Rockford, IL, USA, 2007: 1–7), where 25 μL of the sample was mixed with 200 mL of the working reagent that was prepared at the time, and the mixture was incubated for 30 min at 37 °C. An 8-point calibration curve was also prepared, covering albumin concentrations ranging from 25 to 2000 μg/mL, in addition to a reagent blank as recommended by the manufacturer. The ThermoScientific™ Multiskan™ microplate spectrophotometer (Thermo Scientific, Rockford, IL, USA) was used to perform agitation, incubation, and reading of the absorbances at 562 nm.

### 2.16. SDS-PAGE and Western Blotting

For the electrophoresis, 100 μg of total protein was loaded in 7.5% polyacrylamide gels mounted on the BioRAD electrophoresis system (Mini PROTEAN^®^ Hercules, CA, USA), adding 500 mL electrophoresis buffer solution (192 mM Glycine, 25 mM Tris-base, pH 8.8, 0.1% SDS). The PageRuler™ Plus molecular weight marker (Thermo Scientific, Rockford, IL, USA) was used. Proteins were transferred to BIO-RAD nitrocellulose membranes (Hercules, CA, USA) using the transfer buffer (25 mM Tris-base, 192 mM glycine, 0.04% SDS, and 20% methanol). After the transfer was complete, the nitrocellulose membranes were washed with TTBS (0.2 M Tris-base, 8% NaCl, and 0.1% Tween-20), then blocked with 5% skim milk in TTBS for one h, and finally incubated at 4 °C overnight with the primary antibodies against the NADPH oxidase subunits; NOX2 (gp91^phox^) and p67^phox^ (BD Biosciences, Franklin Lakes, NJ, USA), p22^phox^ (Santa Cruz Biotechnology, Santa Cruz, CA, USA), p47^phox^ (Upstate, Lake Placid, NY, USA), as well as glyceraldehyde-3-phosphate dehydrogenase (GAPDH; Santa Cruz Biotechnology, Santa Cruz, CA, USA), diluted in blocking solution. The following day, the membranes were washed with TTBS and incubated at room temperature for 3 h with anti-mouse-IgG or anti-rabbit-IgG antibodies conjugated with radish acid peroxidase (HRPS, Santa Cruz Biotechnology, Santa Cruz, CA, USA) as secondary antibodies. The membranes were washed with TTBS and developed through the chemiluminescence method with peroxidase substrate using SuperSignal^®^ West Femto Maximum Sensitivity Substrate (Thermo Scientific, Rockford, IL, USA) for a minute. Then the membranes were exposed to radiographic film (PIRCE, USA), developed using Hunt Auto X-Ray fixative and developer liquid (Fujifilm Hunt Chemicals, Allendale, NJ, USA), until the band pattern was visible. Finally, the films were washed, dried at room temperature, and scanned. The densitometry analysis of the bands was performed using the ImageJ program version 1.49 (Wayne Rasband, National Institutes of Health, USA).

### 2.17. Computational Docking

To investigate the potential binding of the tested compounds with the binding interface of p47^phox^ and p22^phox^, molecular docking simulations were conducted using the crystal structure PDB code 7YXW. For this purpose, the cytoplasmic tail of p22^phox^ in the complex was removed, and the contact zone between the SH3A and SH3B domains was defined as the binding pocket for the evaluated compounds. Initially, the structures of **C2**, **C3**, **C4**, **C5**, **C6**, **C9**, **C10**, and **C14** were constructed with 2D Sketcher, from Maestro, Schrödinger, LLC, New York, NY, USA, 2020-2. Next, both ligand and p47^phox^ structures were prepared for docking using Maestro’s LigPrep and Protein Preparation Wizard softwares (Protein Preparation Wizard, Schrödinger, LLC, New York, NY, USA, 2020-2) (www.schrodinger.com, accessed on 9 May 2023). In each case, three-dimensional geometries were created, appropriate bond orders were assigned, ionization states were generated at pH = 7.0 ± 2.0, hydrogen atoms were added, and hydrogen bonds were optimized. The Receptor Grid Generation tool implemented in Glide (Schrödinger) was used to delimit the search space of the ligands to be docked. The center of coordinates of the resulting grid (20 × 20 × 20 Å^3^) was set at the residues W193, W204, F209, D243, E244, W263, M278 and Y279 of the SH3A-SH3B binding interface. Docking calculations were performed with the Glide XP precision mode, which generated 10 potential binding conformations for each compound. These conformations were analyzed with the Schrödinger LLC pose viewer. The best docking poses were chosen using several criteria, such as XP GScore docking energy, interaction with key protein residues, and conformation of the ligand in the active site versus the p22^phox^ pose. Finally, ligand-protein interactions with a high frequency of occurrence between docked ligand poses and p22^phox^ binding site residues were captured using the default parameters predefined in the Canva (Schrödinger) interaction fingerprint calculation panel.

### 2.18. Statistical Analysis

All the experiments were performed in triplicate, using three different cell preparations, and the data were reported as mean ± s.e.m. For the determination of the IC_50_ of the indole heteroaryl-acrylonitriles, concentration-response curves (0.05 to 50 μM) were constructed. IC_50_ values were given as the mean ± standard deviation for at least three independent assays. The data were analyzed using one- and two-way ANOVA. A value of *p* < 0.05 was considered statistically significant. Statistical analyses were performed using the program GraphPad Prism^®^ version 6.00, San Diego, CA, USA.

## 3. Results

### 3.1. Compounds

**Figure 1:**
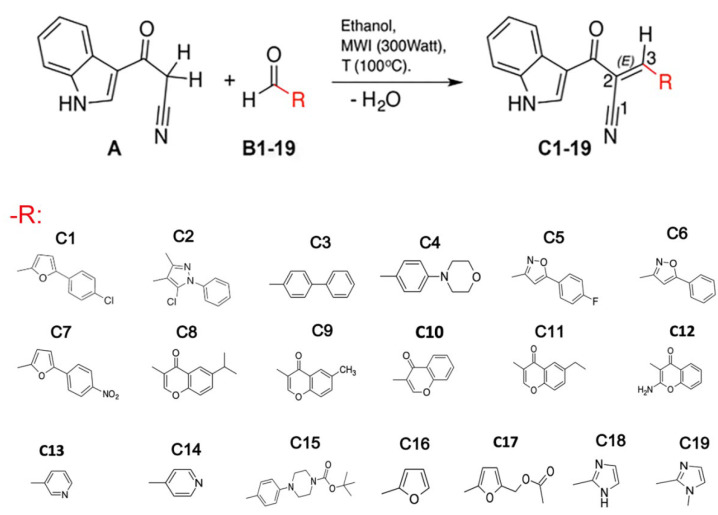
Structure of the (*E*)-2-(1*H*-Indole-3-ylcarbonyl)-3-heteroaryl-acrylonitriles studied. The reaction of cianoacetil-indole (**A**) with different aldehydes (**B**) to form the compounds studied (**C1**–**C19**) is depicted. The R group for the 19 compounds (**C1**–**C19**) produced is presented.

Nineteen (*E*)-2-(1*H*-indol-3-ylcarbonyl)-3-heteroarylacrylonitriles were synthesized (C1-C19) using 3-(cyanoacetyl) indole as a precursor with different heteroaryl aldehydes in ethanol through the microwave-assisted Knoevenagel condensation reaction, as described previously [40,49] (Figure 1).

### 3.2. NADPH Oxidase Inhibitory Activity of Heteroarylacrylonitrile Derivatives in HL-60 Cells

**Figure 2:**
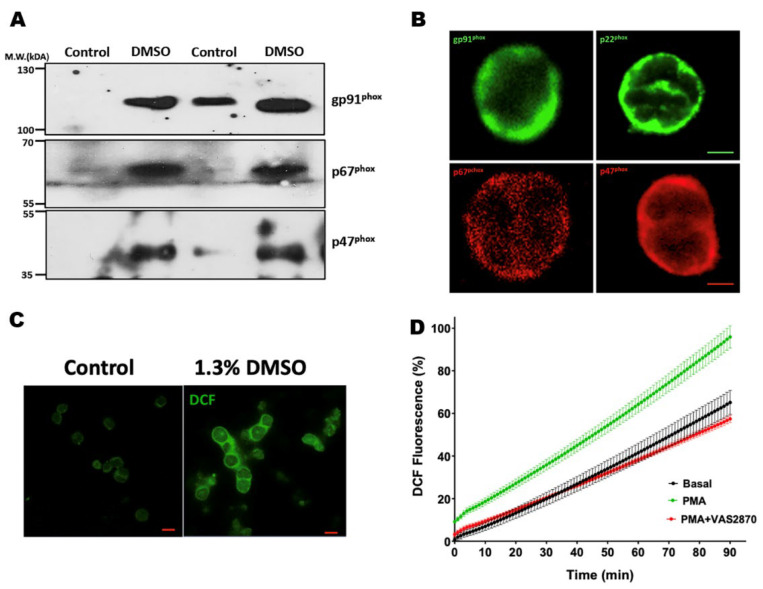
Differentiation of HL-60 cells into neutrophils, and assessment of reactive oxygen species (ROS production). (**A**) Western blot for gp91^phox^, p67^phox^, and p47^phox^ NOX2 subunits. The lanes indicate homogenates of HL-60 cells untreated and HL-60 cells differentiated to neutrophils by treatment for 7 days with 1.3% DMSO. (**B**) Confocal microscopy image of the subunits of NOX2 present in HL-60 cells-differentiated and treated with phorbol 12-myristate 13-acetate (PMA, 0.8 µM). Green indicates the subunits gp91^phox^ and p22^phox^, and red indicates the cytoplasmic subunits p47^phox^ and p67^phox^. (**C**) Representative confocal microphotographies of ROS production in untreated and HL-60 cells differentiated to neutrophils by treatment for 7 days with 1.3% DMSO, activated with PMA 0.8 µM. (**D**) Graph depicting ROS production assessed by 2′,7′-dichlorofluorescein diacetate (DFC-DA) oxidation by fluorimetry. The graph indicates the change in DCF fluorescence intensity in the cells under basal conditions (not stimulated), treated with PMA, and PMA in the presence of VAS2870. Bars indicate 10 μm.

We established a working platform to evaluate the potential NOX2 inhibitory activity of the new indole heteroarylacrylonitrile derivatives (Figure 2). For this, we used HL-60 cells that were differentiated into neutrophils by treatment with DMSO. Under these conditions, HL-60 cells expressed the components of NOX2, including gp91, p22^phox^, p47^phox^, and p67^phox^. Next, the cells were loaded with probe 2′,7′-dichlorofluorescein (DCF), and the fluorescence of the oxidation product of the probe was monitored. The technique was settled, establishing that 100% of the fluorescence induced by ROS is the signal produced by differentiated cells by DMSO and activated with phorbol myristate acetate (PMA) 0.8 µM, which promotes the activation of NOX2 by inducing the phosphorylation of the cytosolic subunits p47^phox^ and p67^phox^ by direct activation of protein kinase C (PKC), followed by translocation of the cytosolic subunits and activation of NADPH oxidase [1,8]. Inhibition control was performed on activated cells by PMA in the presence of VAS2870 (20 µM), a known inhibitor of NOX2 (Figure 2). The basal condition (not activated) was performed with cells in the absence of PMA and the synthesized compounds.

**Figure 3:**
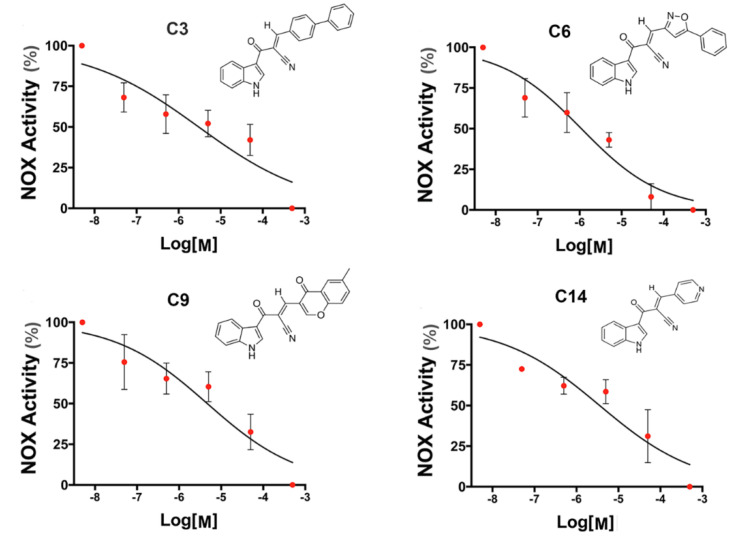
Concentration-response curves of(*E*)-2-(1*H*-Indole-3-ylcarbonyl)-3-heteroaryl acrylonitriles with NOX2 inhibition properties. The ability to inhibit NOX2-derived ROS production was assessed by monitoring in real time the fluorescence of 2′,7′-dichlorofluorescein (DCF) in HL-60 differentiated cells stimulated with phorbol 12-myristate 13-acetate (PMA, 0.8 µM) and treated with increasing concentrations of heteroaryl acrylonitriles. IC_50_ values are indicated for each compound. N = 4 independent experiments for each treatment.

With this paradigm, we assessed the ability to inhibit the NOX-derived ROS production of the nineteen indole heteroaryl acrylonitriles. For this, PMA- activated HL-60 cells were incubated with increasing concentrations of the heteroaryl-acrylonitrile compounds (0.05 μM to 50 μM). With these concentration–effect curves, the IC_50_ for each compound was determined (Table 1). Of the series analyzed, compounds **C3**, **C6**, **C9**, and **C14** exhibited concentration-dependent inhibition of NOX2 (Figure 3).

### 3.3. Cytotoxicity

**Figure 4:**
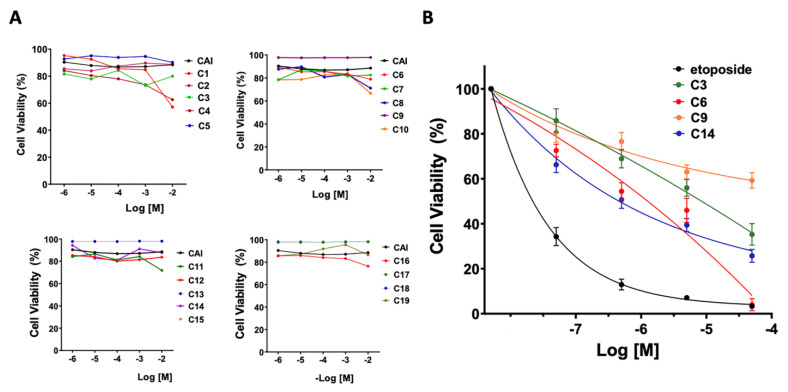
Assessment of cytotoxicity of indole heteroaryl-acrylonitriles in neutrophil-differentiated HL-60 cells. (**A**); Assessment of viability (%) of HL-60 cells incubated with indole heteroaryl-acrylonitrile derivatives for 24 h. CAI; 3-cyanoacetylindole. (**B**), cell viability of HL-60 cells (%) after incubation for 72 h compounds **C3**, **C6**, **C9**, and **C14**. Etoposide was used to control cytotoxicity.

The potential cytotoxicity of the indole heteroaryl acrylonitriles was evaluated by determining the metabolic reduction of 3-(4,5-dimethylthiazol-2-yl)-2,5-diphenyltetrazole bromide (MTT) using HL-60 cells (Figure 4). First, cells were incubated with the compounds at concentrations ranging from 1 µM to 10 mM for 24 h. It was verified that at 24 h, most of the indole heteroaryl-acrylonitriles had no significant cytotoxic effects. Only at 10 mM cell viability found to be reduced to 60% for **C1**, **C4**, **C8**, and **C10**.

Next, we evaluated the cytotoxicity of the compounds after 72 h of incubation, using etoposide as a toxicity control and increasing concentrations of the compounds between 0.01 μM and 100 μM. It was found that the average cellular viability for the evaluated compounds was between 55.4 and 75.9%. In addition, compounds **C6**, **C3**, **C14**, and **C9** were found to be non-cytotoxic in the concentration range evaluated. Although neutrophil-differentiated HL-60 cells had a viability of about 5% against a 100 μM concentration of compound **C6**, maintained for a period of 72 h, this compound had a cytotoxicity of about 55% at the concentration of 1 μM corresponding to the IC_50_ value.

### 3.4. Activity of NADPH Oxidase by Chemiluminescence

**Figure 5:**
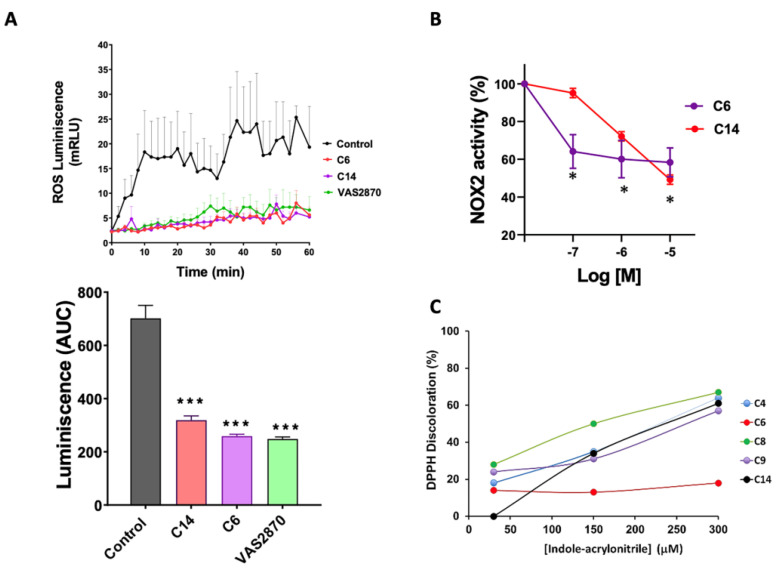
NOX2 inhibition evaluated in HL-60 cell membrane preparations. (**A**). NADPH Oxidase activity assessed by chemiluminescence. Upper panel, a graph showing NOX2 activity as relative units of luminescence per minute, measured by lucigenin chemiluminescence in membrane preparations of HL-60 cells treated with phorbol 12-myristate 13-acetate (PMA) alone (0.8 µM), PMA + VAS2870 (20 µM), and compounds **C14** and **C6** (both 50 µM). Lower panel: graph depicting the area under the curve (AUC) for each treatment. (**B**). NOX2 activity evaluated by cytochrome C reduction in membrane preparations. The bar graphs show the percentage of NOX2 activity in membrane preparations of HL-60 cells treated with increasing concentrations of compounds **C14** and **C6**. (**C**). Assessment of antioxidant capacity of the indole heteroaryl-acrylonitrile compounds. The graph shows the antioxidant capacity of the new acrylonitrile derivatives, expressed as percentage of the discoloration of DPPH in a concentration range between 30–300 µM. N = 3 independent experiments for each treatment. *, *p* < 0.05 vs. control; ***, *p* < 0.0001 vs. control.

A second series of experiments was performed using cell membrane homogenates of differentiated HL-60 cells pretreated with PMA as a NOX2 activator, VAS2870 as an inhibitor, and compounds **C6** and **C14** as an alternative measurement of ROS production in a cell-free system (Figure 5). Here we evaluated the time course of NADPH oxidase activity as relative units of luminescence per minute, measured by lucigenin in membrane preparations obtained by cryo-fracture (Figure 5A). In this condition, compounds **C6** and **C14** were found to significantly reduce ROS production compared to the control condition.

### 3.5. NADPH Oxidase Activity by Reduction of Cytochrome C

A third series of experiments using cryogenic HL-60 membrane homogenates was performed, in which cytochrome C reduction was evaluated as an alternative measurement of superoxide production in a cell-free system (Figure 5B). In this condition, it was found that compounds **C6** and **C14** reduced superoxide production in a concentration-dependent manner.

Next, we tested whether these compounds may be reducing ROS levels through direct radical scavenging activity. For this, the antioxidant capacity of the new compounds was determined by the discoloration of the violet radical 2,2-diphenyl-1-picrylhydrazyl hydrazide (DPPH). As shown in Figure 5C, compounds **C4**, **C6**, **C8**, **C9**, and **C14** showed antioxidant capacity only at very high concentrations, while compound **C14** presented an antioxidant capacity of 12% in the concentration range (30–300 μM). The rest of the compounds did not produce discoloration in the assay (not shown). Therefore, it can be assumed that in the pharmacological range studied (up to 50 μM), the antioxidant effects of compounds **C6** and **C14** are minimal.

### 3.6. ROS Production Assay by the Conversion of 2′,7′-Dichlorofluorescein Diacetate (DCF-DA) in Cardiomyocytes Isolated from Mdx Mice

**Figure 6:**
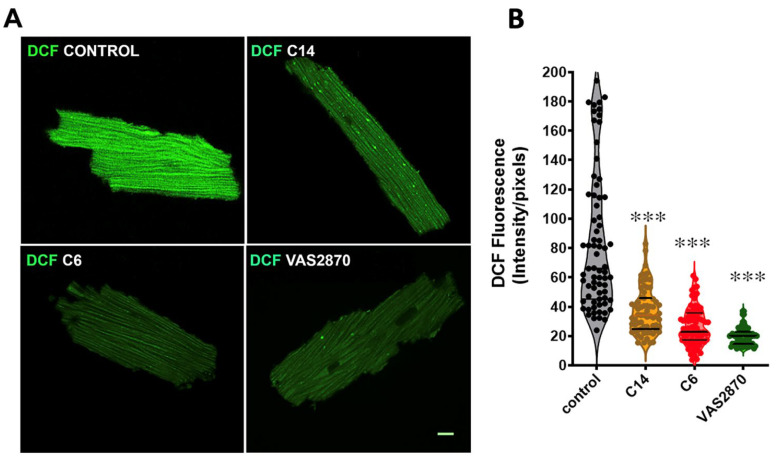
Inhibition of NOX2-derived ROS production in cardiomyocytes from *mdx* mice. (**A**). Representative confocal microphotographies of isolated *mdx* cardiomyocytes loaded with the ROS-sensitive probe DCF (green) fluorescent probe and treated with the compounds **C6**, **C14** (both 1 µM) and VAS2870 (20 µM) as a control. The bar indicates 10 μm. (**B**)**.** Violin graphs showing the fluorescence intensity of DCF from individual myocytes, measured as intensity per pixel. Cells for each condition were obtained from 3 mdx mice’s hearts. One-way ANOVA, ***, *p* < 0.0001, vs. control.

As an additional and independent protocol to evaluate the ability of the indole heteroayl-acrylonitriles to inhibit NOX2-derived ROS production, we used isolated cardiomyocytes from *mdx* mice, a model of Duchenne muscular dystrophy. This model exhibits increased ROS production as a result of NOX2 overexpression [50,53]. To monitor DCF oxidation, we performed a confocal microscopy analysis (Figure 6). It was observed that ROS derived from NOX2 are reduced following treatment with VAS2870 (20 µM) as control of inhibition. Similar trends were observed after treatment with the compounds **C6** and **C14** (1 µM each), resulting in a significant decrease in fluorescence intensity compared to the *mdx* myocytes under the control condition. These results showed that in different cell types, compounds **C6** and **C14** are able to reduce NOX2-derived ROS production.

### 3.7. Translocation of the p47^phox^ Cytosolic Subunit by PMA Stimulation

**Figure 7:**
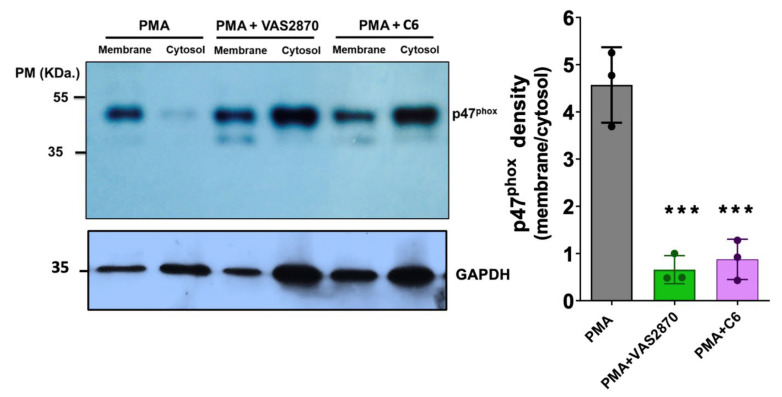
Evaluation of p47^phox^ membrane translocation in HL-60 cells Western blot analysis of phorbol 12-myristate 13-acetate (PMA)-stimulated p47^phox^ translocation in HL-60 cell homogenates. Left panel representative Western blot showing the translocation of p47^phox^ from the cytosol to the membrane after treatment with PMA (0.8 µM) alone, PMA plus VAS2870 (20 µM), and PMA plus compound **C6** (50 µM). Right panel: graph showing the membrane/cytosol ratio of p47^phox^ after the pharmacological treatments. *** *p* < 0.0002, n = 3 independent experiments.

Next, we aimed to investigate the possible mechanisms of inhibition of these compounds. For this, we evaluated p47^phox^ translocation from the cytosol to the membrane in HL-60 differentiated cells, treated with PMA to promote NOX2 activation, and incubated with VAS2870 and compound **C6** (Figure 7). Subsequently, membranes and cytosolic fractions were obtained by cryo-fracture, and the presence of p47^phox^ was analyzed in these fractions by Western blot. Our results showed that compound **C6** (50 μM) significantly reduced the translocation of p47^phox^ to the membrane after stimulation with PMA, similar to VAS2870 (20 µM).

### 3.8. Molecular Docking of ***C6*** and ***C14*** with p47^phox^

The inhibition of translocation of p47^phox^ suggested that compounds **C6** and **C14** may act as inhibitors by competing with p22^phox^ for the binding site on activated p47 ^phox^.

**Figure 8:**
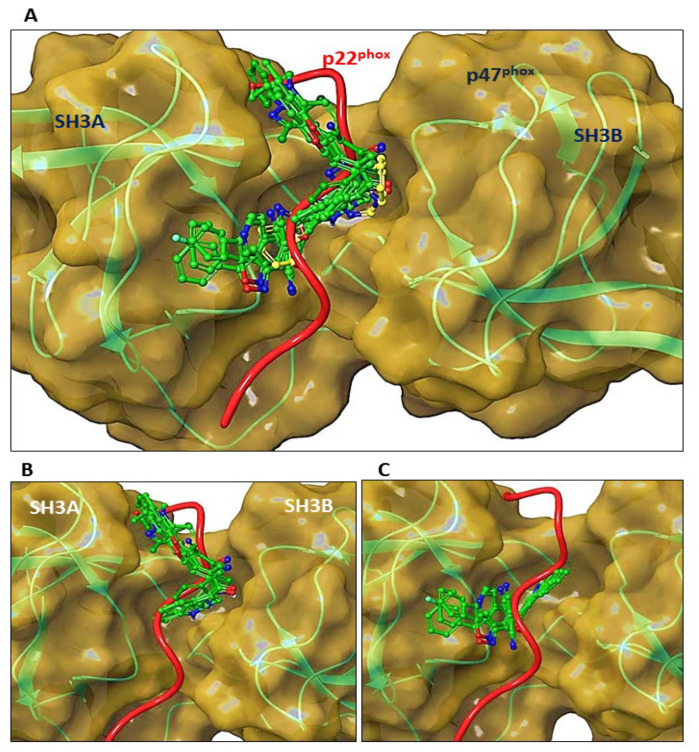
Molecular docking of indole heteroaryl-acrylonitriles in the groove of activated p47^phox^. (**A**): Comparison of the binding mode of **C2**, **C3**, **C4**, **C5**, **C6**, **C9**, **C10**, and **C14** ligands (green) with the p22^phox^ tail (red) and VAS2870 (yellow) in the binding interface of activated p47^phox^. (**B**): binding mode of the less active ligands (**C2**, **C4**, **C9**, and **C10**). (**C**): binding mode of the most active ligands **C3**, **C5**, **C6**, and **C14**.

Based on these findings, we analyzed the binding modes of the indole heteroaryl-acrylonitriles and VAS2870 with p47^phox^ using in silico molecular docking (Figure 8). To evaluate potential interactions of compounds **C2**, **C3**, **C4**, **C5**, **C6**, **C9**, **C10**, and **C14** (all of them with IC_50_ ≤ 10 µM for NOX2 activity) at the binding interface of p47^phox^ and p22^phox^, molecular docking simulations were performed at the groove that formed between the SH3A and SH3B domains of p47^phox^ and the region that accommodates the cytosolic tail of p22^phox^, and binding scores were obtained (Table 2).

These analyses showed that the compounds were allocated into two different segments of the p47^phox^ binding region to p22^phox^, which is established between the SH3A and SH3B domains: in the upper segment or in the deeper segment of the binding groove: **C2**, **C4**, **C9**, and **C10**. While **C3**, **C5**, **C6**, and **C14**, as well as VAS2870, penetrated the inner part of the groove.

**Figure 9:**
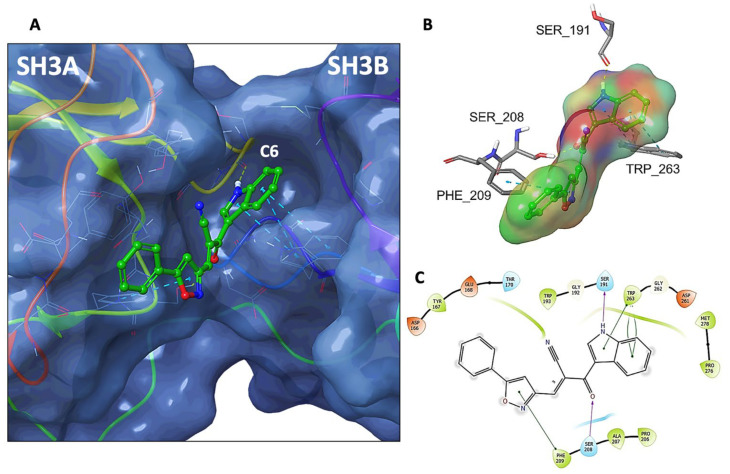
(**A**) Overview of compound **C6** (CPK representation) within the p47^phox^ binding pocket (molecular surface) extracted from docking simulations (C6 carbon atoms in green). (**B**) 3D scheme of p47^phox^-C6 interaction in a surface representation (colored by partial atomic charge). Hydrogen bridge interactions are represented by dashed yellow lines and π-π staking by dashed light blue lines. (**C**) 2D interactions diagram of the docking-generated pose for compound **C6**, highlighting the key main interactions with amino acid residues of p47^phox^. Green lines depict π-π staking, magenta lines indicate hydrogen bonds.

In this region of activated p47^phox^, C6 established interactions with the residues Ser^191^ and Ser^208^ (hydrogen bonds) and with Phe^209^ and Trp^263^ (π-π stacking) (Figure 9). The same was observed for **C14**, except that this molecule established a π-π interaction between Tyr^167^ (instead of Phe^209^) and the pyrimidine group (Supplementary material). Therefore, in agreement with the experimental assays, computational predictions support the association of compounds **C6** and **C14** with p47^phox^ at the binding interface with p22^phox^.

**Figure 10:**
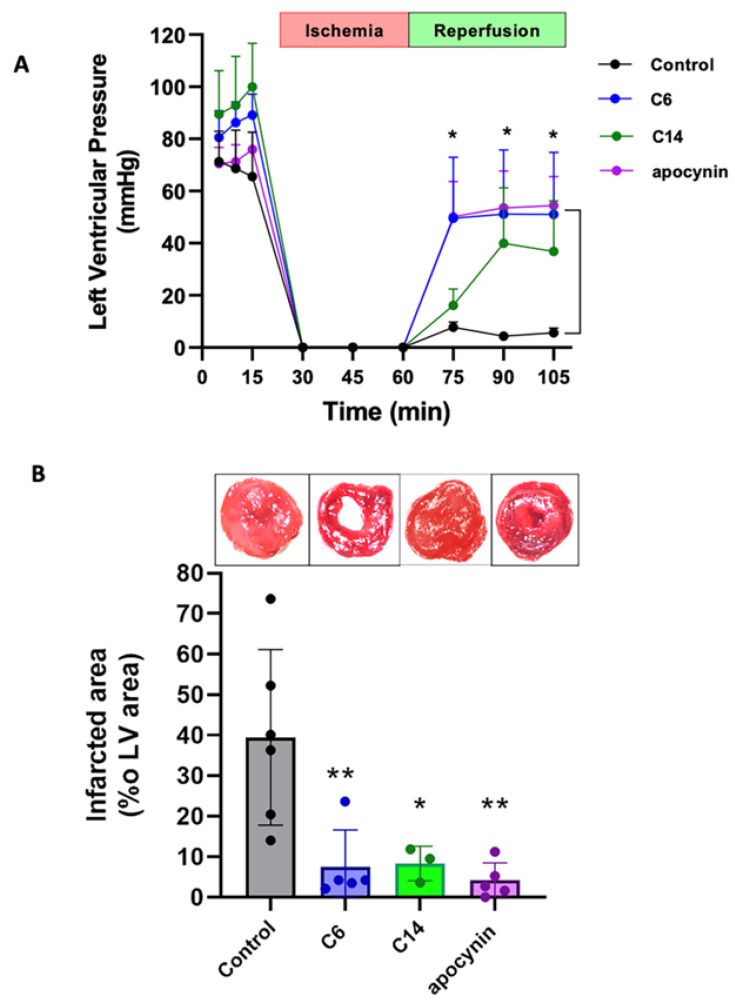
Impact of indole heteroaryl-acrylonitriles on cardiac damage induced by ischemia-reperfusion. (**A**) Graph depicting the values of ventricular developed pressure in control conditions and in the presence of compounds **C6** and **C14**, both 1 µM; and apocynin (100 µM). (**B**) Evaluation of compounds **C6**, **C14**, and apocynin on infarct size in the protocol of cardiac ischemia-reperfusion. The numbers of hearts used: control n = 6; **C6** n = 5; **C14** n = 3; apocynin n = 5. * *p* < 0.05, ** *p* < 0.01 vs. control, one- or two-way ANOVA.

Finally, we evaluated the indole heteroaryl-acrylonitriles in a model of cardiac ischemia-reperfusion since ROS play a central role in the pathophysiology of the damage induced by reperfusion [54], particularly NOX-derived ROS [55,56]. For this, we isolated rat hearts and mounted them in a Langendorff perfusion system, as previously described [51]. We used the perfusion of apocynin as a reference cardioprotective agent, as it has been recently reported [57]. As can be observed in Figure 10A, during the baseline period of perfusion (min 0–15), neither of the compounds altered the perfusion pressure or cardiac rhythm (not shown). After reperfusion, both compounds increased left ventricular pressure (LVP) compared to the controls (*p* < 0.05). Finally, cardiac damage was evaluated as an infarcted area in the hearts submitted to the ischemia-reperfusion protocol (Figure 10B). This analysis showed that both compounds reduced the infarcted area compared to control hearts, similar to apocynin.

## 4. Discussion

Here, we have evaluated a series of indole heteroaryl-acrylonitrile derivatives as potential inhibitors of the NADPH oxidase NOX2. Our results demonstrated that two of these compounds exhibited NOX2 inhibitory activity, as evidenced by their ability to reduce ROS production in three different assays while showing minimal ROS scavenging activity. These compounds were also effective in pathophysiological conditions, such as the damage observed in the heart after being subjected to ischemia-reperfusion. Furthermore, our data reveal that the mechanism of action of these compounds involves a disruption of the interaction between p22^phox^ and p47^phox^, which is required for NOX2 activation. This suggests that indole heteroaryl-acrylonitrile derivatives may have specificity for NOX2 inhibition and hold promise as potential therapeutic agents for diseases involving NOX2 overactivity.

The catalytic core of the NOX2 complex is formed of a transmembrane protein containing two heme molecules and the binding sites for NADPH and FAD (gp91^phox^) [5]. The transmembrane protein p22^phox^ is also part of this complex [2]. In order to catalyze ROS production, NOX2 requires activation, which is a multiple-step process involving phosphorylation and translocation of cytosolic subunits such as p67^phox^, p40^phox^, p47^phox^, and the small GTPase Rac, which is a member of the Rho family of small GTPases [6].

For NOX2 enzymatic activation, the interaction between a groove formed by a tandem Src homology 3 (SH3) domain of p47^phox^ and a proline-rich region of p22^phox^ is critical [6]. This complex has been crystallized and characterized both in terms of structure and function. Furthermore, the crystal structure of the p47^phox^ tandem SH3 domain with the proline-rich peptide is known [9]. The activation of NOX2 requires conformational changes in the cytoplasmic complex to allow the assembly of the heterohexameric enzyme at the membrane. The binding of the p47^phox^-p67 ^pho^ -p40^phox^ complex to the membrane-bound cytochrome b558 (gp91^phox^ and p22^phox^, both transmembrane proteins) is supported by an interaction between the SH3 tandem domains of p47^phox^ and the cytoplasmic tail of p22^phox^. The crystal structure of the tandem SH3 domains of p47^phox^ in complex with a peptide derived from the C-terminal tail of p22^phox^ (amino acids 149–166) revealed that both SH3A and SH3B domains cooperate to mediate this interaction [6]. In the absence of the SH3B domain, the SH3A domain can also contribute to the complex formation. This has been demonstrated by its ability to interact with the proline-rich peptide. However, the interaction between p47^phox^ and p22^phox^ subunits is significantly improved through additional contacts made by SH3B. In the self-inhibited p47^phox^ structure, SH domains act together in tandem to generate a large domain (SuperSH) that interacts with the peptide by simultaneously contacting conserved residues from both domains. Once on the membrane, additional contacts occur between p47^phox^ and cytochrome b55, which could help properly position the p67^phox^ subunit or induce a conformational change within the cytochrome.

Oxidative stress, which describes an imbalance between the synthesis of ROS and antioxidants, has been implicated in the generation of numerous pathologies, including endothelial dysfunction and cardiovascular diseases such as hypertension and heart failure [58,59,60]. ROS generation can occur through different sources, such as enzymatic systems, including NADPH oxidases (NOX) [54,56]. Five NOX isoforms have been described, which, despite the structural similarity, differ in the mechanism of activation, tissue distribution, and physiological function. On the other hand, there are currently a variety of substances that have been investigated in order to inhibit the production of ROS dependent on NOX [19,22,61], such as NOX2 [62]. However, potent and isoform-specific inhibitors of NOXs have not been identified yet, making them an excellent target for the development of new compounds that can decrease or inhibit the production of ROS in a particular NOX isoform, i.e., NOX2 and NOX4 [63].

We synthesized a series of heteroaryl-acrylonitrile compounds (**C1**–**C19**) [40,49] and identified two of them with NOX2 inhibitory capacity. This opens up possibilities for the development of selective NOX2 inhibitors by utilizing structure-based studies to analyze the interactions between indole-heteroarylcrylonitriles and p47^phox^.

The complexity of NOX isoform structures, particularly the membrane-associated core subunits [2,64], has made the development of NOX inhibitors challenging. The search for isoform-specific small molecule inhibitors has faced even more hurdles [22,65]. However, targeting the protein-protein interactions of the subunits that activate the enzymatic complex has emerged as a promising strategy for achieving specificity, as these interactions differ among NOX2 isoforms [58,66,67,68].

For NOX2, targeting the protein-protein interactions of the subunits that lead to the NOX2 active complex, particularly the interaction between p22^phox^ and p47^phox^ has potential benefits for the development of inhibitors, as it may lead to isoform-specific inhibition while avoiding off-target effects on other NOX isoforms [69]. The first compound described to interrupt this interaction was Ebselen, which was reported to prevent the translocation of p47^phox^ [67]. Nevertheless, Ebselen has been found to have multiple biological targets, and it has also been reported to form a covalent adduct with p47^phox^ [66], raising concerns about its specificity as a NOX2 inhibitor.

Another molecule proposed to interact with the p22^phox^-p47^phox^ complex is LMH001 [58], but this notion has been challenged recently since this compound was shown to be unstable and unable to effectively interfere with p22^phox^-p47^phox^ interactions [70]. Finally, CPP11G and CPP11H, two bridged tetrahydroisoquinolines, have been suggested to interfere with this protein–protein interaction, but there is no biophysical evidence of this interaction [71]. In addition to small molecules, peptides and peptide-mimetics have also been shown to interfere with the p22^phox^-p47^phox^ complex [72,73]. Interestingly, these compounds interact with a series of amino acid residues in the groove of p47^phox^ that also include Trp^263^ and Ser^208^, two residues that were shown to be critical for bidding in the cases of **C6** and **C14**.

*Limitations of the study.* In this work, we studied systematically the properties of indole heteroaryl-acrylonitriles as NOX2 inhibitors. Nevertheless, we did not evaluate their specificity over the other NOX isoforms. Those studies remain to be performed. Furthermore, it remains to analyze the actual values of the physicochemical interactions between the compounds and p47^phox^. It is also worth mentioning that VAS2870, a compound extensively used as a reference in this study for its NOX2 inhibitory properties, is not isoform-selective and exhibits off-target effects [74,75].

## 5. Conclusions

These novel compounds represent promising candidates for further investigation and experimental studies focused on the inhibition of NADPH oxidase, particularly NOX2 inhibition. The insights gained from this study can be used to guide the design of new NOX inhibitors with improved efficacy and provide a foundation for future drug discovery efforts targeting this important complex.

## Figures and Tables

**Table 1:** IC_50_ values for NOX2 inhibition of(*E*)-2-(1*H*-Indole-3-ylcarbonyl)-3-heteroaryl acrylonitriles in HL-60 cells.

Compound	IC_50_ (µM)
**C1**	21.5 ± 0.3
**C2**	7.2 ± 0.3
**C3**	3.0 ± 0.3
**C4**	7.1 ± 0.4
**C5**	6.2 ± 0.4
**C6**	1.1 ± 0.2
**C7**	68.1 ± 1.2
**C8**	17.1 ± 0.2
**C9**	4.7 ± 0.3
**C10**	9.4 ± 0.3
**C11**	19.9 ± 0.2
**C12**	21.1 ± 0.2
**C13**	16.3 ± 0.2
**C14**	3.6 ± 0.2
**C15**	30.4 ± 0.3
**C16**	82.7 ± 0.2
**C17**	19.7 ± 0.2
**C18**	51.3 ± 0.2
**C19**	69.3 ± 0.2

**Table 2:** Values for docking energy scores (*E*)-2-(1*H*-Indole-3-ylcarbonyl)-3-heteroaryl-acrylonitriles.

Compound	XP GScore (kcal/mol)
**C2**	−4.078
**C3**	−6.080
**C4**	−5.138
**C5**	−7.389
**C6**	−7.576
**C9**	−1.612
**C10**	−6.195
**C14**	−6.897
VAS2870	−5.482

## Data Availability

Data will be made available upon reasonable request.

## Supplementary Materials

The following supporting information can be downloaded at: https://www.mdpi.com/article/10.3390/antiox12071441/s1.
