# Supplementary material for: New NADPH Oxidase 2 Inhibitors Display Potent Activity against Oxidative Stress by Targeting p22phox-p47phox Interactions
Source: Antioxidants (Basel). 2023 Jul 18;12(7):1441. doi: 10.3390/antiox12071441 (PMC10376059; doi:10.3390/antiox12071441)
Supplement: Supplementary file 1 [file antioxidants-12-01441-s001.zip › antioxidants-2416793-supplementary.pdf]

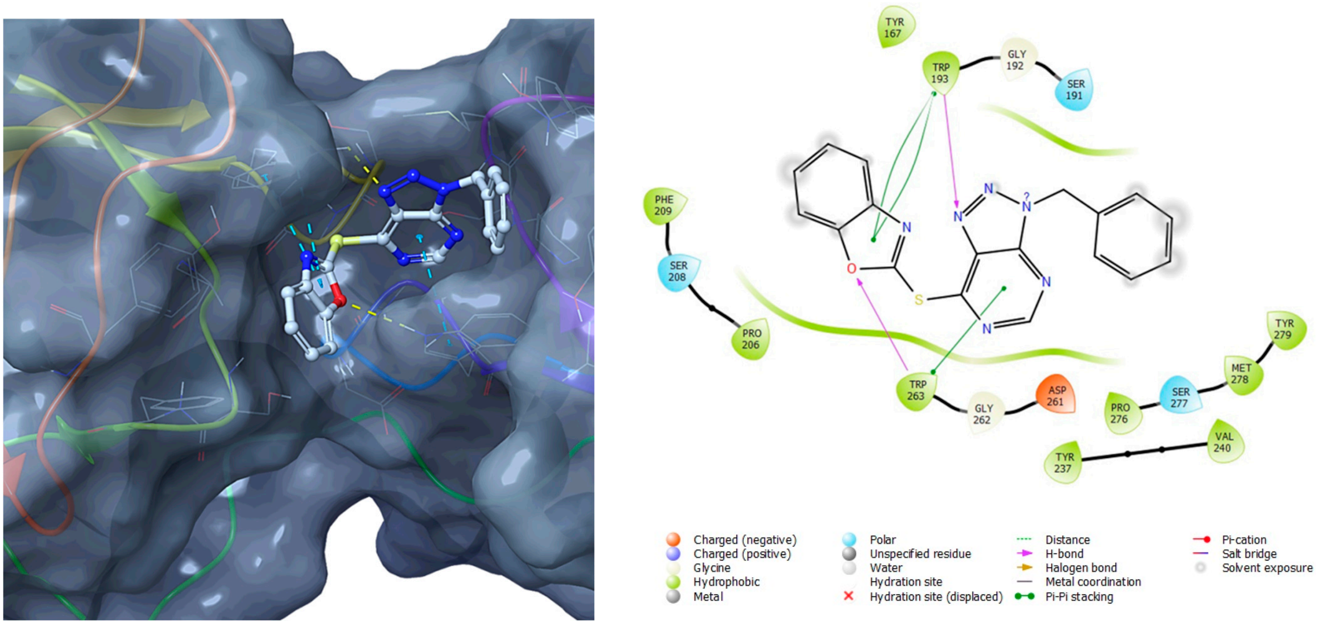

Figure S1. Overview of VASP2870 docking within the p47phox binding pocket. Left, CPK representation of docking-generated pose for VAS2870. Right, 2D diagram of the docking generated pose of VAS2870, showing main interactions with p47phox aminoacidic residues.

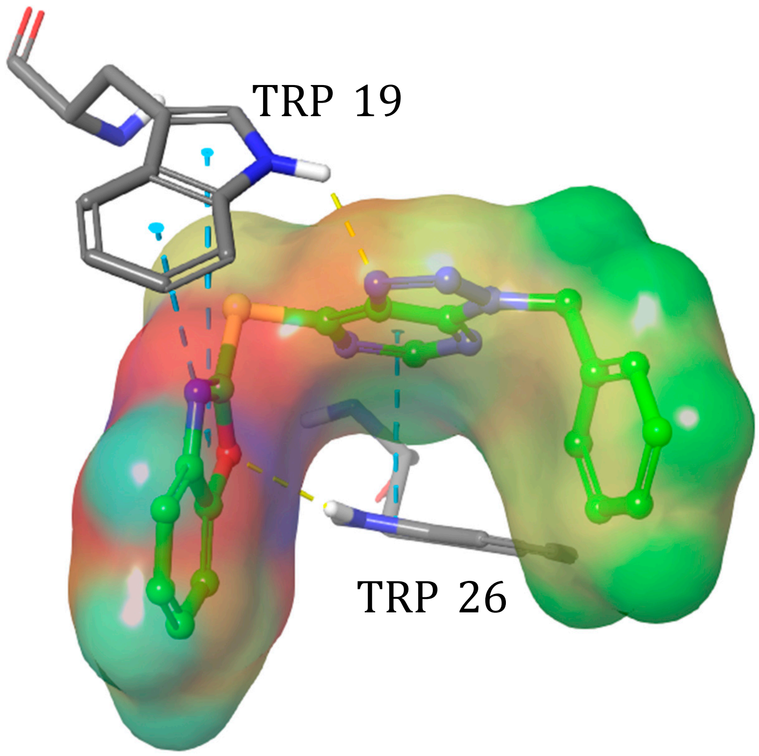

Figure S2. 3D scheme of protein-ligand interaction colored by atomic charge for VAS870 and p47phox. The figure depicts the interactions between VAS2870 and p47phox residues Trp193 and Trp263. Yellow dashed lines represent hydrogen bridge and blue lines π-π stacking.

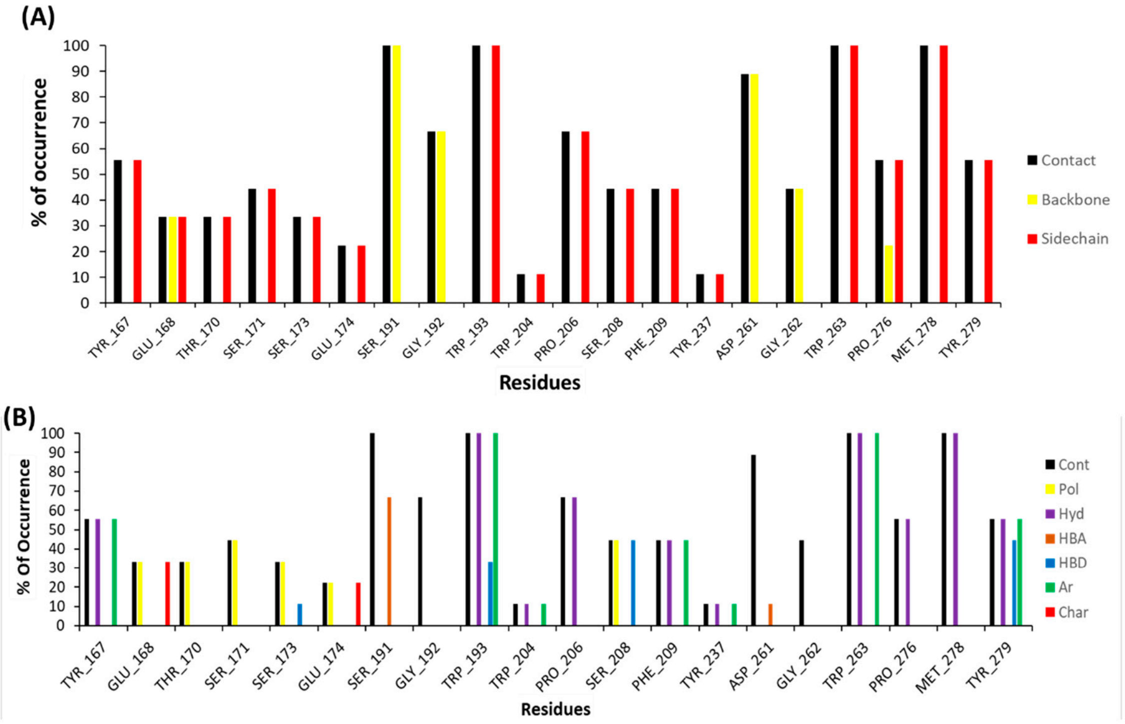

Percent occurrence of interaction types at the P47phox-ligand binding interface for complexes obtained by molecular docking. Percent occurrences of Contacts, interactions with the backbone of residue, and interactions with the side chain of residue for the nine complexes **(A)**. The percentages of occurrences of chemical interactions: Contacts, **Cont**; polar, **Pol**; hydrophobic, **Hyd**; where the residue is acceptor, **HBA**; where the residue is donor, **HBD**; aromatic **Ar**, and electrostatic with charged groups **Char** for the nine complexes **(B)**.

Figure S3. Main residues of p47phox involved in interactions with the indole heteroaryl-acrylonitriles studied. A., percentage of ocurrence of contact between backbone or sidechain residues and the compounds. B, Type of interactions between p47phox binding pocket resides and the indole heteroaryl-acrylonitriles studied.

P47Phox/C14

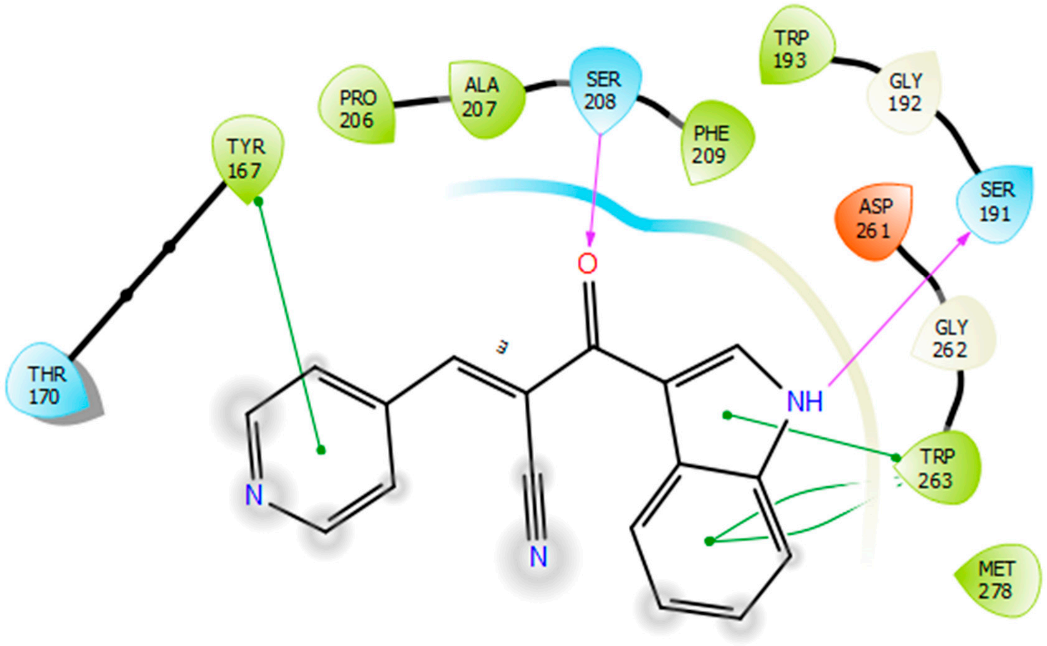

Figure S4. 2D diagram of the docking generated pose of C14 inside the p47phox binding pocket, showing main interactions with p47phox aminoacidic residues. Green lines depict π-π interactions. Purple lines indicate hydrogen bond.
